# Rapid Detection of Viable Microorganisms Based on a Plate Count Technique Using Arrayed Microelectrodes

**DOI:** 10.3390/s130708188

**Published:** 2013-06-26

**Authors:** Avneet Bajwa, Shaoqing Tim Tan, Ram Mehta, Behraad Bahreyni

**Affiliations:** 1 Alberta Health Services, Calgary, AB T2N 2T9, Canada; E-Mail: abajwa@sfu.ca; 2 School of Mechatronic Systems Engineering, Simon Fraser University, Surrey, BC V3T 0A3, Canada; E-Mail: ttan@sfu.ca; 3 PBR Laboratories Inc., Edmonton, AB T6E 0P5, Canada; E-Mail: rmehta@pbr.ca

**Keywords:** biosensor, impedimetric detection, foodborne pathogens, microorganisms, plate count, bacterial colony, lab-on-chip

## Abstract

Development of a miniaturized biosensor system that can be used for rapid detection and counting of microorganisms in food or water samples is described. The developed microsystem employs a highly sensitive impedimetric array of biosensors to monitor the growth of bacterial colonies that are dispersed across an agar growth medium. To use the system, a sample containing the bacteria is cultured above the agar layer. Using a multiplexing network, the electrical properties of the medium at different locations are continuously measured, recorded, and compared against a baseline signal. Variations of signals from different biosensors are used to reveal the presence of bacteria in the sample, as well as the locations of bacterial colonies across the biochip. This technique forms the basis for a label-free bacterial detection for rapid analysis of food samples, reducing the detection time by at least a factor of four compared to the current required incubation times of 24 to 72 hours for plate count techniques. The developed microsystem has the potential for miniaturization to a stage where it could be deployed for rapid analysis of food samples at commercial scale at laboratories, food processing facilities, and retailers.

## 1. Introduction

The presence of foodborne pathogens and toxins can lead to severe health consequences in humans. In the United States alone, one in six Americans suffer from a foodborne illness every year, which results in nearly 130,000 hospitalizations as well as 3,000 deaths [1]. On the other hand, bacterial contamination in processed food has led to many large scale recalls of beef, turkey, spinach, nuts, and even pet food [2–5]. Such massive recalls result in huge financial losses as well as the disposal of valuable food. With the concentration of food-processing plants, there is an increasing need for the development of novel and faster methods to detect microorganisms and ensuring that the processed food complies with the relevant standards of quality. Consequently, many governments are taking steps to strengthen food safety monitoring regulations [1]. In addition to establishing preventive measures, more testing and monitoring is required to improve food safety. Therefore, there is an increasing need for systems that can quickly test a food sample for bacterial contamination at low cost. Such systems may then be deployed at the food processing plants, food safety laboratories, and even retailers.

There has been a significant increase in the field of electrical bio-detection techniques for pathogen detection with the promises of reduced cost and simplified signal processing [6–10]. Impedimetric sensing relies on the measurement of the electrical properties of the medium around a set of electrodes to detect changes [11–14]. A basic impedimetric sensor is made of a pair of electrodes to monitor the voltage/current relationship for a test medium. A reference signal, often sinusoidal, is used to excite the drive electrode while the amplitude and phase of the current through medium is detected using the other electrode. Impedimetric biosensors are used for various applications including cell culturing [12,15], monitoring of cell migration [16], and pathogen detection [11,17,18]. In many cases, the biosensors are integrated within a microfluidic system for sample handling [15,19,20]. However, the developed systems so far either monitor the direct interactions of individual or few bacteria with biosensors, investigate the changes in electrical properties of the whole medium (e.g., a sample of milk), or a single bacterial colony over time [13,14,17,19–22]. Even though these techniques can provide information on the existence of bacteria in a sample, in most cases they are unable to produce data on the actual number of bacteria in the original sample. Additionally, many of the proposed systems require a change of testing techniques for pathogens [19,23,24]. From a commercial adaptation perspective, it is highly advantageous if the proposed techniques comply with the standard methods in the industry [25].

On occasion, a sample needs to be tested to see whether a specific pathogen, such as *Escherichia coli* or *Salmonella*, is present. In such cases, one needs to resort to tests that can detect a specific type or strain of bacterium [26,27]. Polymerase chain reaction (PCR) has been typically employed to selectively detect the bacteria [27,28]. However, capital costs for automated PCR systems are quite high. On the other hand, the required consumables are also expensive. Additionally, PCR suffers from some practical limitations, such as cross contamination and the inability to determine whether the detected pathogen was viable or not at the time of sampling, among others.

Techniques that only detect and quantify live bacteria in a sample are widely used across the industry for evaluation of food and water samples. This is partly due to the high costs and long times associated with specific tests (e.g., PCR). On the other hand, in many cases, the total number of bacteria, regardless of the type, is also an indicator of the quality and suitability for human consumption. Plate count technique is widely employed in food industry in order to indiscriminately quantify the population of bacteria in a food sample [25,29]. The process starts with liquefying the sample (if needed) followed by filtering to remove solid particles and dilution. The sample is then spread over a growth medium. Over a 24 to 72 hour incubation time, each bacterium in the sample forms a colony, which is then detected through visual inspection. Several dilution steps of the original sample are often necessary in order to have isolated colonies that can be easily counted. The total number of bacteria in the original sample can then be estimated by extrapolating the number of colonies knowing the number of dilution steps. In its basic form, plate count technique provides a rough estimate on the number of the microorganisms but does not discriminate against their types. It is however possible to selectively target specific types or strains of bacteria by adding inhibitors to the growth medium. The required incubation time is a major shortcoming of plate count technique as a late positive detection can be quite costly. On the other hand, the need for visual inspection limits the number of samples that can be processed at a given facility.

In this paper, we describe our design and implementation of a microsystem for automation of plate count technique. The system comprises an array of impedimetric biosensors which are integrated into the design of a micro-incubation chamber and the required interface and multiplexing electronics. The developed system significantly reduces the incubation time from the current required time of 24 to 72 hours to less than 6 hours. The electrical interface of the system makes it possible to automate the detection process, which can increase the throughput of samples through a testing facility. Finally, the whole setup can be realized as a compact bench-top system, which can be used at various facilities. Sample preparation and procedures for testing are nearly identical to the established industrial protocols while the system provides results in a fraction of the current times. On the other hand, the experimental setup and biochips can be manufactured inexpensively, paving the way for adaptation of the proposed system.

## 2. Experimental Section

### 2.1. Microfabrication of Sensor Array and Incubation Microchamber

**Figure 1.**
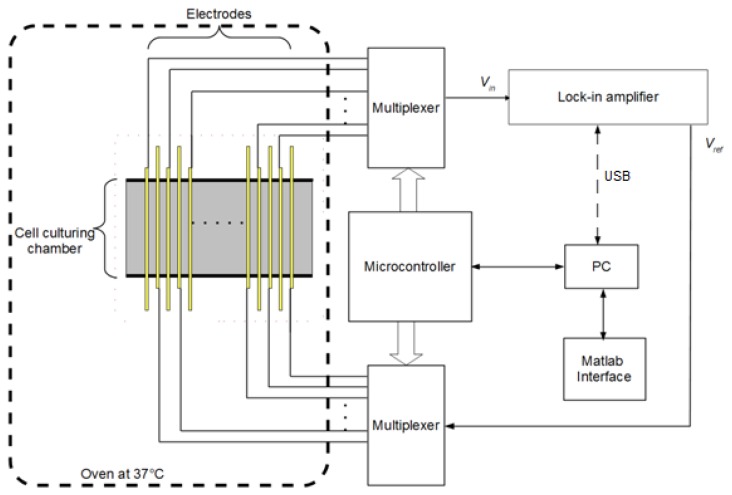
Block diagram of pathogen detection system.

**Figure 2.**
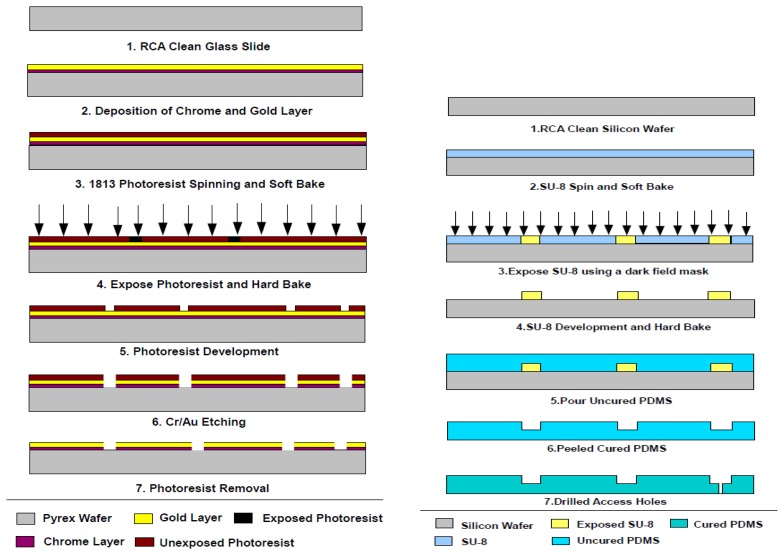
Fabrication process for biosensor electrodes (**Left**) and the PDMS microchamber (**Right**). The two chips are bonded together to form the culturing microchamber.

Figure 1 illustrates the conceptual drawing of the system, which is composed of an array of biosensors with an integrated incubation chamber, a multiplexing circuitry, measurement electronics, and a computer to monitor and control the system and store the data. The biosensors are made of arrays of closely spaced gold electrodes patterned on a glass substrate. Different electrode configurations were examined to produce a sensitive and repeatable measurement considering that the thickness of the culturing medium above the electrodes was about 1 mm. Too small a gap between the electrodes makes detection of changes at the surface of the culturing medium difficult. On the other hand, a wide gap results in loss of spatial resolution and sensitivity. Final electrodes were designed to be 100 μm wide and 2.5 cm long with an inter-electrode gap of 100 μ m . The electrodes were made 100 nm of sputtered gold layer above a 10 nm film of sputtered chrome as an adhesion layer. The metal layers were patterned using conventional photolithography and wet etching processes on 75 mm × 75 mm glass slides. Prior to metallization, glass slides were RCA cleaned to rid of any surface contamination. The fabrication process is illustrated in Figure 2.

To fabricate the microchamber, polydimethylsiloxane (PDMS) prepolymer and its curing agent (Sylgard 184 Silicone Elastomer Kit, Dow Corning, Midland, MI, USA) were thoroughly mixed in a 10:1 weight ratio. The pre-polymer mixture was poured onto the master mold and was degassed in a desiccator for 1 hour to remove any air bubbles in the mixture. The PDMS was cured for 5 hours at 80 °C in a conventional oven. After curing, the PDMS replicas were peeled off from the master mold and were cut to size (2 cm by 1 cm) with a standard surgical steel blade. Access holes were punched in the reservoirs using gauge 14 blunt needles. The PDMS chamber was then bonded to the electrode chip after surface activation of the glass substrate in oxygen plasma.

### 2.2. Interface Electronics and Data Collection

**Figure 3.**
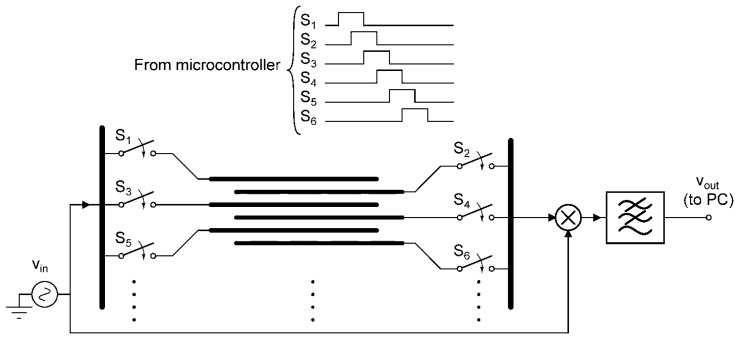
Simplified schematic of the multiplexing and measurement electronics. The timing signals for the switches are shown at the top.

The fabricated chip was then connected to the interface electronics. The goal is to precisely measure the electrical properties of the medium between microelectrodes. This can be achieved through using a reference signal that is applied to one electrode while measuring the output signal from a counter-electrode. To improve the noise performance and sensitivity of the system, a synchronous demodulation circuit is used to detect minute variations in relative signal amplitude over time. If needed, this also presents the user with the relative phase information of the sensed signal which can be employed to further improve performance. To reduce the circuit complexity, a multiplexing network was used in order to scan through all microelectrodes and monitor the impedance levels across the chip. The timing signals for the multiplexing networks were provided by a microcontroller (MSP420G2452 from Texas Instruments, Dallas, TX, USA). Once the data for a pair of microelectrodes was ready, it was read and stored on a personal computer. Figure 3 illustrates a simplified diagram of the interface electronics including the microelectrode array, switches, and synchronous demodulator. The personal computer also communicated with the microcontroller to initiate the tests. The control software, developed using Matlab^®^, provided a graphical user interface and file IO.

### 2.3. Growth Medium Preparation and Application

**Figure 4.**
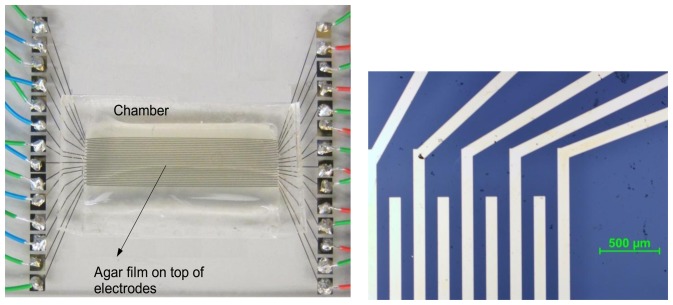
Biosensor chip with agar film prior to culturing (**left**) and close-up of the electrodes (**right**).

To conduct a test, the biosensors within the incubation chamber were covered with a culturing medium. The culture medium was prepared through dissolving three capsules of Lysogeny Broth medium from MPBiomedical (Solon, OH, USA) and 1.8 g of agar (powder) from Anachemia (Lachine, QC, Canada) in 120 mL of deionized (DI) water in an autoclaving flask. The mixture was thoroughly mixed and autoclaved at 121 °C for 55 minutes. The electrode surface and chamber walls were processed in the microfabrication cleanroom. They were then transferred to a lab with proper biosafety requirements and the liquefied agar was poured into the chamber using a serialized pipette. The liquid volume was selected such that it covered the entire microchamber uniformly. It was left undisturbed to solidify and form a film with a thickness of about 1 mm. The final biosensing chip is shown in Figure 4.

## 3. Results and Discussion

**Figure 5.**
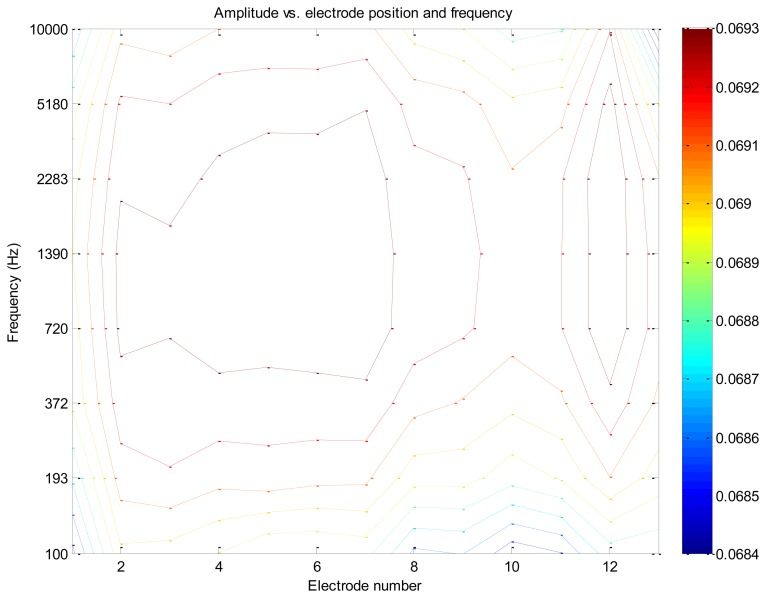
Contour plot indicating presence of cells at electrode pairs 2, 3, 4, 5, 6, 7 and 12.

Initial experiments were carried out to test the sensitivity and response time of the electrical biosensor. To reduce the experiment time and be able to conduct the test in normal lab environment, initial experiments were conducted using commercial yeast. When suspended in sugar water, yeast normally incubates at room temperature after 4 minutes. The system was capable of capturing the yeast activity such as their initial growth and multiplication as well as the slowdown in growth due to the limited food supply. Figure 5 shows a contour plot of the system response in presence of yeast on the growth medium over a range of signal frequencies. As can be seen, for medium frequencies (*i.e.*, from about 300 Hz to 5 kHz) the system has the strongest response. In general, it is necessary to evaluate the frequency response of the system, comprising the electrodes and growth medium, prior to selecting an operating frequency. However, as can be seen in this figure, there is usually a range of signal frequencies that one may employ and still have strong signals.

To assess the system performance against food- or water-borne pathogens, a cell solution containing *Escherichia coli* DH5α bacteria was prepared. A single colony of bacteria was transferred from a culture place into a test tube containing 10 mL of 0.1% peptone water. This solution was diluted in three steps down to obtain 1:100 ratio with respect to the original sample. This cell solution was found to produce single colonies over the culturing medium and was used for the experiments. The prepared cell solution could be used for up to a week if stored at 4 °C . To test the system, 400 μL of the diluted cell solution was dropped at the center of the chamber using a pipette and evenly spread using an autoclaved cell-spreading rod. The cell solution was allowed to dry for about 15 minutes at room temperature. It was then transferred to an oven at 37 °C with near saturation level of relative humidity for culturing.

**Figure 6.**
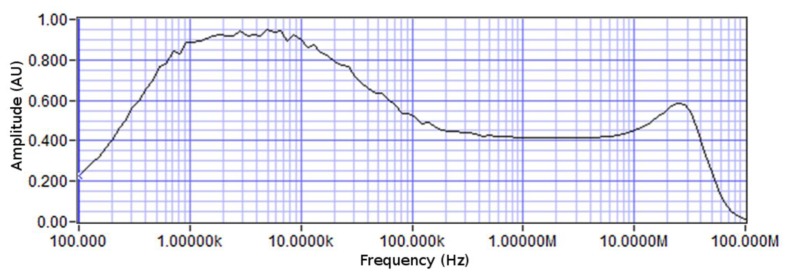
Spectral response of the impedance of electrode array with growth medium.

**Figure 7.**
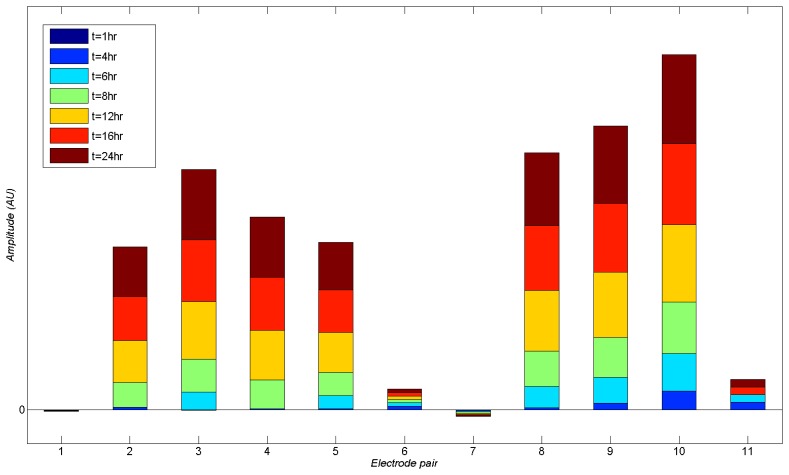
Incremental increase in signal amplitudes read from different electrode pairs. Strong signals indicate presence of bacterial colonies.

In addition to the chip containing the bacteria, a control biochip with no bacteria at its surface was used in order to provide a benchmark for the tests. Different test signal frequencies ranging from 100's of Hz to several MHz were used for monitoring of the electrical properties of the medium. The impedance spectrum of the chip is shown in Figure 6. At low frequencies, the capacitive transduction is not efficient, resulting in relatively weak measured signals. On the other hand, at high frequencies, the parasitics from wiring and enclosures affect the measurement more strongly. Therefore, the chosen operating frequency was always between 1 kHz and 10 kHz. During the system operation, the control electronics scans across the chip continuously and measures the data from different biosensors using the multiplexing network. Figure 7 illustrates the incremental changes in signal levels as time progressed over a 24 hour incubation time. As can be seen, after 16 hours of incubation, there is a significant difference between measured signals from different sensors as an indication of bacterial activity over a number of biosensors (*i.e.*, biosensors 2 and 3 and 6 to 8). However, as early as 6 hours from the starting of the experiment, one can observe a meaningful change in signal amplitude corresponding to the presence of bacteria.

**Figure 8.**
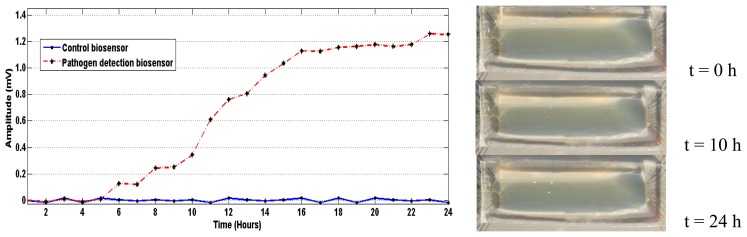
Signals from biosensor #7 on the test biochip underneath a bacterial colony and the same biosensor on the control chip. Pictures to the right show the growth of bacterial colony over time.

Figure 8 shows the changes in signal levels from a biosensor directly beneath a bacterial colony over time. Signals from the control biochip are also presented for comparison. As can be seen, presence of bacterial colonies can be detected as early as 6 hours from the beginning of incubation. However, if one relies on the visual inspection, the colonies are barely visible after 10 hours of incubation and are generally incubated for at least 24 hours (for *E. coli*) before counting bacterial colonies. It can therefore be seen that the proposed system shortens the measurement time by at least a factor of four.

## 4. Conclusions and Outlook

Plate count technique is a relatively simple method for detection of presence of viable pathogens in a food sample. This technique is widely used across the industry to estimate the number of the pathogens in a food or water sample. A main shortcoming of the technique is the extended amount of time that is required for incubation, which is typically between 24 and 72 hours. Relying on highly sensitive biosensors, the system presented in this work can reduce the incubation time significantly and alleviate the need for visual inspection. The system readings are differential (*i.e.*, are compared against the initial test conditions), and therefore, initial non-uniformities in sample preparation or dispersion do not affect the system performance. The presented microsystem composed of an array of biosensors, a multiplexing circuitry, timing and control electronics, and data capture and storage was developed for rapid detection of foodborne microorganisms. A major benefit of the proposed method is its compatibility with the current procedures and standards in industry for food- or waterborne pathogen detection. Moreover, not only the system can reduce the incubation time by a factor of 4 to 12, it also makes it possible to automate the process and provide information on the number and location of bacterial colonies. Effectiveness of the system was demonstrated through early detection of yeast and E. coli bacteria. In all cases studied, the system could identify visible colonies. Future work on this project will concentrate on devising methods to lower the overall system cost, in particular, by replacing the gold electrodes with another suitable material. Moreover, the effects of various growth mediums and inhibitors on system performance will be investigated. An automated signal processing mechanism will be needed to enable automatic counting of the colonies based on the number and strength of signal variations across the sample.
